# Intrinsic ADE: The Dark Side of Antibody Dependent Enhancement During Dengue Infection

**DOI:** 10.3389/fcimb.2020.580096

**Published:** 2020-10-02

**Authors:** Rohan Narayan, Shashank Tripathi

**Affiliations:** ^1^Department of Microbiology & Cell Biology, Indian Institute of Science, Bengaluru, India; ^2^Centre for Infectious Disease Research, Indian Institute of Science, Bengaluru, India

**Keywords:** dengue, pathogenesis, antibody dependent enhancement (ADE), extrinsic ADE, intrinsic ADE

## Abstract

Dengue fever is an *Aedes* mosquito-borne illness caused by any one of the four different dengue virus (DENV) serotypes (1–4) and manifests in the form of symptoms ranging from mild or asymptomatic to severe disease with vascular leakage, leading to shock, and viral hemorrhagic syndrome. Increased risk of severe disease occurs during secondary infection with a virus serotype distinct from that of prior dengue infection. This occurs by antibody dependent enhancement (ADE) of infection, wherein sub-neutralizing antibodies against the virus particles opsonize dengue virus entry via formation of immune complexes that interact with fragment crystallizable gamma receptors (FcγR) on monocytes, dendritic cells, and macrophages. The ADE phenomenon has two components: Extrinsic and Intrinsic ADE. While extrinsic ADE contributes to enhanced virus entry, intrinsic ADE results in heightened virus production by inhibition of type1 interferon and activation of interleukin-10 biosynthesis, thereby favoring a Th2 type immune response. Intrinsic ADE has greater contribution in enhancing Dengue replication as compared to extrinsic ADE. Detailed elucidation of intrinsic ADE during secondary dengue infection can increase our understanding of DENV-pathogenesis and aid in the development of host-targeting antivirals. Here we review literature focusing on intrinsic factors contributing to severe dengue pathology and suggest possible avenues for further research.

## Introduction

Dengue fever results from infection with any of the four DENV serotypes via the bite of infected *Aedes* sp. mosquitoes. They are single stranded positive sense RNA viruses belonging to the family *Flaviviridae*. A lipid bilayer membrane surrounds the virus particle and contains the envelope (E) and membrane (M) proteins embedded on the surface. The DENV E protein binds to specific receptors on susceptible cells to gain entry, followed by which the viral RNA genome uncoats and begins replication in the cytoplasm (Guzman et al., 2016). Translation of viral proteins also ensues and virus assembly occurs in the endoplasmic reticulum before being transported through the trans-Golgi network and released extracellularly as mature virus particles (Wilder-Smith et al., 2019).

The disease is widespread in tropical and sub-tropical regions of the world and although a vaccine exist for this disease, its use has not been approved in all countries (Aguiar and Stollenwerk, 2018). As per estimates for the year 2013, about 58.4 million symptomatic dengue cases were reported globally, which amounted to US$8.9 billion (Shepard et al., 2016). A study by Murhekar et al. estimated the total number of Dengue infections in India during the year 2017 to be 12,991,337 (Murhekar et al., 2019; Wilder-Smith and Rupali, 2019).

Whilst dengue fever can manifest as an asymptomatic or mild febrile illness, a more severe form of the disease ensues during secondary infection whereby increased vascular permeability and thrombocytopenia causes gastrointestinal hemorrhage and plasma leakage that results in Dengue Hemorrhagic fever (DHF) and Dengue shock syndrome (DSS) (Halstead, 2015). During secondary dengue infection by a heterologous DENV serotype, antibody response in humans is primarily directed against the virus serotype from previous infection(s) and the antibodies thus produced are often non-neutralizing against the heterologous serotype compared to the original infecting serotype from primary infection. The phenomenon is termed original antigenic sin and was first described in Dengue by Halstead et al. (1983). These sub-neutralizing antibodies in turn aid in ADE of dengue infection by catalyzing virus entry via interactions with FcγR on the cell surface (Wilder-Smith et al., 2019).

It should be noted that original antigenic sin in the context of dengue fever may refer to enhancement of DENV pathogenesis by either sub-neutralizing antibodies against the infecting serotype during secondary infection or due to aberrant B and T cell responses targeted against the DENV serotype from primary infection (Rothman, 2011). Severe dengue can also occur during primary DENV infection in infants born to dengue immune mothers. In this case, the initially protective antibody titers decline over the first year after birth and leads to increased infection due to ADE (Libraty et al., 2009).

## Extrinsic vs. Intrinsic ADE

Two types of ADE have been defined, namely extrinsic and intrinsic ADE. Extrinsic ADE, as its name suggests, refers to phenomena that are extrinsic to mononuclear phagocytes, like enhanced rates of receptor interaction and internalization of virus-immune complexes. In fact, extrinsic factors were thought to be solely responsible for the adverse effects of dengue ADE associated pathogenesis, until studies on Ross River Virus (RRV) revealed a different scenario. Incubation of RRV infected cells with anti-virus IgG resulted in ADE mediated persistent productive infection of macrophages for prolonged time periods, brought about by innate immune suppression (La Linn et al., 1996; Halstead, 2015). This phenomenon is called intrinsic ADE, that involves modulation of innate immune effectors by internalized virus-immune complexes to favor increased replication and release (Halstead, 2015). In other words, intrinsic ADE increases the “burst size” of infected cells i.e., virus release from an infected cell, and extrinsic ADE enhances infected cells mass (Flipse et al., 2016a). Here we focus on intrinsic ADE during dengue infections and the myriad ways by which this phenomenon enhances DENV replication and release.

## Innate Immune Responses During Primary Dengue Infection

Severe pathogenicity associated with secondary dengue infection due to intrinsic ADE occurs primarily by evasion of host innate immune responses. A proper understanding of immune response occurring canonical/primary dengue infection is vital to properly appreciate the immune-evasion mechanisms associated with intrinsic ADE. Canonical DENV entry occurs via receptor-mediated endocytosis and recognition of invading pathogen is first detected by pathogen recognition receptors (PRRs). While Toll like receptor (TLR)−3 and TLR-7 detect the virus in endosomes, the low pH dependent escape of viral RNA from these vesicles is recognized by MDA5 (melanoma differentiation-associated gene 5) and RIG-I (retinoic-acid inducible gene 1) (Chen et al., 2008) Activation of both TLR-dependent and independent pathways ensue and promote expression of pro-inflammatory cytokines like IL-12, IL-8, IFN-γ, and IFN-α (Ubol and Halstead, 2010). This coupled with activation of STAT1 by IFNs results in production of nitric oxide (NO) radicals and helps limit DENV replication and spread (Flipse et al., 2016a).

## Intrinsic ADE and Antiviral Immunity

### Evasion of Innate Immunity

The route of DENV entry into cells and early events of virus replication occurring thereafter are critical factors that influence the host response to infection (Flipse et al., 2013; Chan et al., 2019). As opposed to canonical dengue infections, entry of antibody-opsonized DENV during ADE is thought to occur via a phagocytosis-like pathway (Ayala-Nunez et al., 2016) via the cross-linking of activating FcγR present on the surface of monocytes, macrophages and dendritic cells. Such receptor engagement generally triggers a type-1 interferon stimulated gene (ISG) response, but DENV evades this antiviral mechanism by causing the co-ligation of leukocyte Ig-like receptor-B1 (LILRB1), a tyrosine-based inhibition motif-bearing immunoreceptor. This results in the dephosphorylation of spleen tyrosine kinase, a key regulator of FcγR signaling (Chan et al., 2014), thereby inhibiting the type1 ISG response. Yet another study by Chan et al. reported the use of fluorescently labeled DENV-2 to elucidate changes in the host transcriptome as a result of both canonical receptor-mediated endocytosis and the FcγR coupled antibody mediated entry pathway (Chan et al., 2019). Similar viral loads were used to induce virus entry via either of these two pathways and Gene Ontology (GO) terms analysis was used to identify changes in the host transcriptome. Dengue ADE mediated virus entry predominantly caused the enrichment of differentially expressed genes (DEGs) that regulate RNA processing and many of these factors are known to interact with DENV viral RNA (Viktorovskaya et al., 2016). Cross-referencing of this data with previously published genome wide CRISPS/cas9 screens revealed that dengue ADE modulates the transcriptome of monocytes to upregulate genes that are responsible for vesicular transport and mRNA processing. While canonical dengue infection causes downregulation of DEGs responsible for host protein translation, antibody-mediated DENV entry reverses this effect to aid in translation of viral proteins (Chan et al., 2019).

In contrast to canonical dengue infection in the absence of immune-complexes, ADE during secondary infection with heterologous serotype inhibits TLR expression and signaling. The expression of negative regulators of toll like receptor (TLR)-expression such as TANK (TAF family associated NF-κB activator) and SARM (Sterile-alpha and Armadillo Motif containing protein) during dengue ADE results in downregulation of TLR-signaling molecules and TLR-3,4, and 7 expression in DENV infected cells (Modhiran et al., 2010; Flipse et al., 2013). A consequence of this is the abrogation of IRF1 and IRF3 production by activation of NF-κB and Inhibitor of κB kinases (IKKs), respectively, during downstream TLR signaling pathway. The upregulation of TANK signaling pathway facilitates complex formation between canonical IKKs and IKK related kinases which culminates in a shift to TLR signaling via a non-NF-κB pathway instead of the canonical NF-κB mediated signaling (Kawagoe et al., 2009; Clark et al., 2011).

While intrinsic and extrinsic mechanisms of ADE immunopathogenesis has been associated with increased DENV entry in susceptible cells, studies on primary macrophages has shown that this is not always the case (Malavige et al., 2012). The antibody-mediated entry of DENV particles in primary macrophages results in enhanced membrane fusion potential within the endosomes, thereby enhancing virus replication and translation. Since virus entry into these cells remains unaltered, the internalized DENV particles go largely unnoticed by the endogenous interferon pathway and enabling enhanced viral replication during early stages of infection (Flipse et al., 2016a).

### Effects on Virus Replication and Pathogenesis

**Figure 1:**
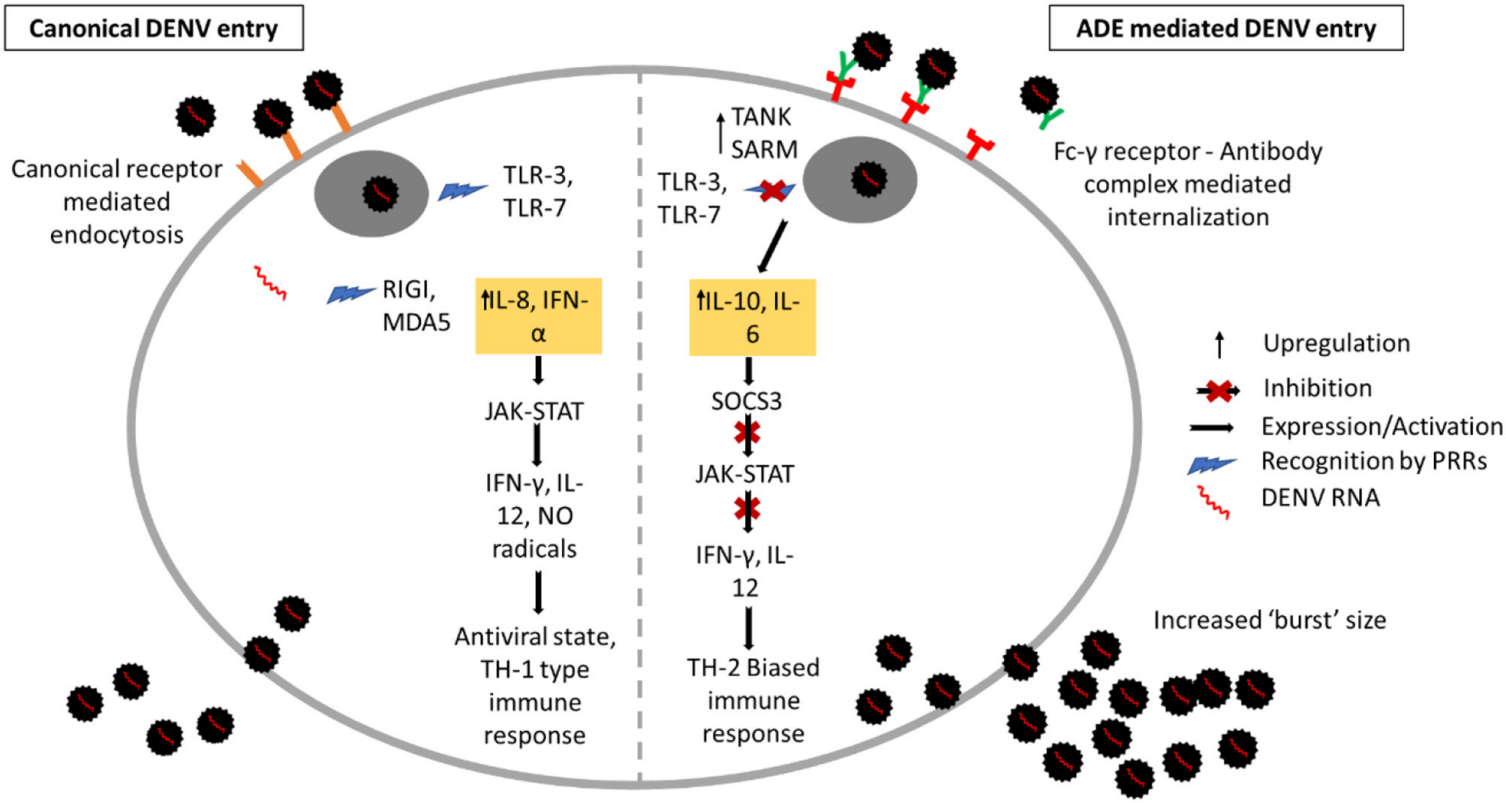
Innate immune response during ADE and non-ADE dengue infection. Canonical non-ADE mediated entry occurs via receptor-mediated endocytosis. Upon entry, the DENV particles are internalized in endosomes and are recognized by the pathogen recognition receptors TLR-3 and 7. Release of viral RNA from endosomes is recognized by RIGI and MDA5 which triggers production of pro-inflammatory cytokines IFN-γ and IL-8. This activates the JAK/STAT pathway resulting in expression of IFN-γ, IL-12, and Nitric Oxide radicals. Virus entry via FcγR-antibody in dengue-ADE caused expression of TANK and SARM which inhibits TLR signaling. Production of anti-inflammatory cytokines IL-10 and IL-6 ensues and expression of SOCS3 as a result inhibits JAK/STAT pathway. This results in inhibition of pro-inflammatory cytokine production and causing a TH-2 biased immune response and increased “burst” size.

Many innate immune effectors have been shown to be modulated in DHF/DSS, out of which the upregulation of IL-10 biosynthesis via intrinsic ADE plays a pivotal role in dampening host mediated innate and adaptive immune responses (Tsai et al., 2013). Dengue induced immunosuppression has been studied *in vitro* by incubating DENV with serum obtained from dengue infected patients, followed by addition of the virus-antibody mixture to THP-1 cells (human monocytic cell line constitutively expressing FcγR). In addition to promoting virus replication, dengue induced ADE was shown to induce a TH2-type immune response as evidenced by increased production of IL10 and IL6. This causes over expression of SOCS3 (Suppressor of cytokine signaling 3 gene) thereby inhibiting the Janus kinase-signal transducer and activator of transcription (JAK-STAT) signaling pathway and production of IFN-γ. A direct consequence of this is the abrogation of NO synthesis, which facilitates increased dengue viral RNA synthesis. Moreover, studies in K562 cells (human chronic myelogenous leukemia cell line) has shown that inhibition of NO synthesis using specific inhibitors increased virus production during dengue-ADE (Flipse et al., 2013). The enhancement of anti-inflammatory cytokine synthesis and subsequent inhibition of Th1-type cytokines IL-12 and IFN-γ during dengue ADE by this intrinsic mechanism produces a Th2-type biased immune response (Chareonsirisuthigul et al., 2007; Ubol et al., 2010). Figure 1 summarizes the effects of DENV infection during ADE and non-ADE conditions.

### Effects on Adaptive Immune Response

A balanced Th-1 and Th-2 type immune response to any infection is crucial for the effective clearance of pathogens (Berger, 2000). While the elicitation of Th-1 type response leads to the production of pro-inflammatory cytokines and increased phagocytic activity, Th-2 type response results in heightened anti-inflammatory cytokine production characterized by type-2 or antibody-mediated immunity. The Th-2 cytokines IL-1, IL-10, and IL-13 promote B-cell proliferation and thereby stimulates antibody production (Spellberg and Edwards, 2001). In the case of dengue-ADE, a skewed Th-2 type immune response serves only to exacerbate the already worse situation by promoting the production of sub-neutralizing antibodies that aid in immune complex-mediated DENV entry into permissible cells (Ubol and Halstead, 2010).

## ADE in Other Viruses

Apart from DENV, the effect of ADE on enhancement of virus pathogenesis has been shown to true in the case of few other viruses. The classic example is that of HIV-1 wherein increased viral RNA and protein synthesis ensues when cells are infected in the presence of HIV-1 specific antibodies as compared to untreated cells (Robinson et al., 1989). Similar results also have been reported for other viral diseases like West Nile fever (Gollins and Porterfield, 1985), Ross River fever (Lidbury and Mahalingam, 2000) feline infectious peritonitis, porcine reproductive and respiratory syndrome (PRRS) and Aleutian disease of mink (Halstead et al., 2010). Enhancement of Zika virus infection in the FcγR positive K562 cells was shown to be enhanced in the presence of DENV specific antibodies (Castanha et al., 2017). This effect was seen in STAT2^−/−^ mouse model, where sera from dengue and West Nile positive patients enhanced Zika virus infection and diseases in FcγR dependent manner (Bardina et al., 2017). Studies done using primary macrophages revealed that Zika virus infection resulted in the downregulation of IFNβ and reactive nitrogen intermediates (Hueston et al., 2017). Large scale epidemiological investigations to closely monitor the clinical relevance of such findings in important. For instance, immune correlates of severe dengue was matched with data from a long term study on Nicaraguan children and it was shown that intermediate levels of dengue antibodies lead to exacerbation of disease compared to low (non-protective) or high (protective) levels of antibodies (Katzelnick et al., 2017). Such studies could also help gain better insights into dengue immunity and rationale of vaccine designs (Waggoner et al., 2016). The modulation of Dengue/Zika pathogenicity by DENV antibodies and the prospects of using therapeutic antibodies for protection against both these diseases has been reviewed elsewhere (Khandia et al., 2018).

More recently, a study employing the use of pseudotyped virus particles has reported that SARS Corona virus entry in permissive cells is enhanced in the presence of neutralizing monoclonal antibodies (MAbs) against Middle East Respiratory Syndrome (MERS) coronavirus spike. Binding of these MAbs with virus particles functionally mimics the virus receptor and mediates entry by causing conformational changes that make the spike protein more prone to undergo proteolytic activation (Wan et al., 2020). With respect to the current COVID-19 pandemic caused by SARS CoV-2 virus (Mitchell, 2020), it is imperative that careful testing and validation of prospective vaccines and MAb-based therapies be done to avoid adverse consequences as a result of coronavirus ADE (Wang and Zand, 2020). A summary of the different animal and/or cell models used, and the specific phenotypes studied in different publications is given in Table 1.

**Table 1:** Summary of the animal models and phenotypes studied in select publications cited in this manuscript.

	**Model used for study**	**Virus and phenotype studied**	**References**
1	Mice–Bone marrow derived macrophages, STAT1^−/−^ mice	DENV serotypes 1,2,3, and 4 replication kinetics, secretion of pro/anti-inflammatory cytokines	Chen et al., 2008
2	Primary human macrophages	DENV2 infection kinetics, virus-macrophage fusion potential, gene profiles, and IFN signature	Chan et al., 2014; Flipse et al., 2016a
3	Human primary monocytes	Entry of fluorescent labeled DENV2 particles in monocytes, transcriptome analysis	Chan et al., 2019
4	THP-1 cell line	DENV2 infectivity, pro/anti-inflammatory cytokine production, NO radicals synthesis	Chareonsirisuthigul et al., 2007
5	K562 cell line	Zika virus PE/243 and DENV-2 16681 strains, ADE of Zika infection	Castanha et al., 2017
6	K562 cell line, Stat2^−/−^ C57BL/6 mice	Zika virus infection and pathogenicity in mice; ADE, neutralization *in vitro*	Bardina et al., 2017
7	Primary human macrophages	ZIKV MR766 strain ADE mediated cytokine production, reactive nitrogen intermediates	Hueston et al., 2017

## Conclusions and Perspectives

Gaining better insights at the genomic level is imperative to truly understand the intrinsic manipulation of host immune responses during dengue ADE. This may be achieved with the use of Genome wide CRISPR/cas9 screens in myeloid cell lines. Generation of such genome wide CRISPR/Cas9 knockout screening libraries can be used to elucidate host intrinsic factors that are indispensable for the modulation of host immune system during dengue infections. Results from such experiments can then be compared with already available data to identify targets for antiviral therapy.

In addition, more focus needs to be addressed toward the use of primary myeloid cells isolated from dengue infected and naïve donors. Host response to dengue ADE can vary depending on the type of cells being studied (Boonnak et al., 2011) and although studies using dendritic cells (Flipse et al., 2016b) and monocytes (Sun et al., 2011) have been reported, it is imperative that DENV research done using lab adapted continuous cell lines are compared with results from primary cells as well in order to get a detailed picture.

Studies on the transcriptional downregulation of antiviral genes associated with intrinsic ADE can be supplemented with proteomics studies to gain more insights into the virus-host interactions during severe dengue fever. Cell-targeting drugs against effectors of intrinsic ADE could be used as a prophylactic treatment option for both adults and infants suffering from severe dengue fever. The application of live imaging studies for the elucidation of non-canonical route of DENV cell entry and early events occurring therein, can aid in the identification of targets for both antiviral and for cell-targeting drugs.

The main theme of this mini review is the intrinsic immune evasion mechanisms of DENV infection in humans, leading to severe dengue pathogenicity. On that account, we have summarized the relevant literature highlighting the inherent differences between extrinsic and intrinsic ADE and suggest key points for further research.

## Author Contributions

Conceived, review, and editing by ST. Literature review and draft writing by RN. All authors contributed to the article and approved the submitted version.

## Conflict of Interest

The authors declare that the research was conducted in the absence of any commercial or financial relationships that could be construed as a potential conflict of interest.
